# Electrically Triggered Quercetin Release from Polycaprolactone/Bismuth Ferrite Microfibrous Scaffold for Skeletal Muscle Tissue

**DOI:** 10.3390/pharmaceutics15030920

**Published:** 2023-03-11

**Authors:** Musa Ayran, Hatice Karabulut, Kudret Irem Deniz, Gamze Ceren Akcanli, Songul Ulag, Alexa-Maria Croitoru, Bianca-Maria Tihăuan, Ali Sahin, Denisa Ficai, Oguzhan Gunduz, Anton Ficai

**Affiliations:** 1 Center for Nanotechnology & Biomaterials Application and Research (NBUAM), Marmara University, Istanbul 34722, Turkey; 2 Institute of Pure and Applied Sciences, Department of Metallurgical and Materials Engineering, Faculty of Technology, Marmara University, Istanbul 34722, Turkey; 3 Department of Science and Engineering of Oxide Materials and Nanomaterials, Faculty of Applied Chemistry and Materials Science, University Politehnica of Bucharest, 060042 Bucharest, Romania; 4 National Centre for Micro- and Nanomaterials, University Politehnica of Bucharest, 060042 Bucharest, Romania; 5 National Centre for Food Safety, University Politehnica of Bucharest, 060042 Bucharest, Romania; 6 Research Institute of the University of Bucharest—ICUB, 050567 Bucharest, Romania; 7 Research & Development for Advanced Biotechnologies and Medical Devices, SC Sanimed International Impex SRL, 087040 Calugareni, Romania; 8 Department of Biochemistry, Faculty of Medicine, Marmara University, Istanbul 34722, Turkey; 9 Department of Inorganic Chemistry, Physical Chemistry and Electrochemistry, Faculty of Applied Chemistry and Materials Science, University Politehnica of Bucharest, 060042 Bucharest, Romania; 10 Academy of Romanian Scientists, Ilfov St. 3, 050044 Bucharest, Romania

**Keywords:** biomaterials, quercetin, skeletal muscle, electrospinning, electrically drug delivery, antimicrobial activity

## Abstract

Skeletal muscle tissue engineering presents a promising avenue to address the limitations pertaining to the regenerative potential of stem cells in case of injury or damage. The objective of this research was to evaluate the effects of utilizing novel microfibrous scaffolds, containing the compound quercetin (Q), on skeletal muscle regeneration. Morphological test results showed us that the combination of bismuth ferrite (BFO), polycaprolactone (PCL), and Q were bonded and well-ordered with each other, and a uniform microfibrous structure was obtained. Antimicrobial susceptibility testing of PCL/BFO/Q was conducted, and microbial reduction was found to be over 90% in the highest concentration of Q-loaded microfibrous scaffolds with the most inhibitory effect on *S. aureus* strains. Further, biocompatibility was investigated by performing MTT testing, fluorescence testing, and SEM imaging on mesenchymal stem cells (MSCs) to determine whether they could act as suitable microfibrous scaffolds for skeletal muscle tissue engineering. Incremental changes in the concentration of Q led to increased strength and strain, allowing muscles to withstand stretching during the healing process. In addition, electrically conductive microfibrous scaffolds enhanced the drug release capability by revealing that Q can be released significantly more quickly by applying the appropriate electric field, compared with conventional drug-release techniques. These findings suggest a possible use for PCL/BFO/Q microfibrous scaffolds in skeletal muscle regeneration by demonstrating that the combined action of both guidance biomaterials was more successful than Q itself acting alone.

## 1. Introduction

Skeletal muscle tissue, representing around 40–45% of the total mass of the average adult human body, provides forces that aid in voluntary movement, stability, locomotion, and dynamic activities [1,2]. Skeletal muscle tissue comprises modules of tightly and closely packed muscle fibers containing several myoblast-derived multinucleated cells. The extracellular matrix (ECM) includes fibers highly oriented together to form an organised muscular tissue capable of longitudinal contraction [3,4]. The inherent regenerative capacity of skeletal muscle tissue is endowed with remarkable resilience; notwithstanding, traumatic injuries of significant magnitude that occasion grave destruction to the musculature pose a formidable difficulty in terms of their management within the confines of primary healthcare [5]. Due to the restricted mobility of the wounded organ, the slow rate of skeletal muscle regeneration can induce deformation in the spinal structure in addition to the primary injury. This is caused by the absence of resident satellite cells (tissue-specific muscle stem cells) in organs [6]. Recent progress in cell treatment employing myoblasts or muscle-derived stem cells has opened up new therapeutic approaches for regenerating muscle tissue for functional improvement. Cultivated myoblasts have demonstrated some achievements, but may not be appropriate for repairing significant muscular tissue deficiencies [7,8,9]. Tissue-engineering technology has become prevalent as a method to repair injured tissues or organs. Delivery of pre-engineered muscle constructs to injured locations, including cultured myoblasts or muscle-derived stem cells on a matrix scaffold, is the most efficient method for the functional regeneration of muscle [10,11]. The interaction of cells with biomaterial surfaces is thought to be vital in achieving optimal cellular structures for muscle tissue, and knowledge of cellular activities including cell adhesion, proliferation, and migration is required. It is commonly acknowledged that surface properties, including wettability, chemistry, electric charge, and topography of surfaces affect cell adherence and proliferation [12]. A suitable material should be comprised of biocompatible, biodegradable properties and electrical stimulation that allows the native muscle to maintain relaxation and enhance cell growth [13,14,15,16]. Since electrical stimulation plays a critical role in the functionality of skeletal muscles, it is reasonable to investigate the impact of electrical stimulation on skeletal muscle regeneration in vivo, especially considering the presence of neural structures that provide stimulation and activate regulatory feedback systems within myofibers [17]. Langelaan et al. investigated the impact of electrical stimulation on the maturation of muscle cells in vitro, using 2D and 3D culture systems. Specifically, they examined the effect of applied electrical fields on C2C12 muscle monolayers in a 2D culture system and found that this led to a rapid reduction in the availability of nutrients in the surrounding medium. Their study provides valuable insights into the role of electrical stimulation in promoting muscle cell maturation and development in vitro [18]. In addition, it was observed that a significant number of differentiated myotubes exhibited synchronised contractility when subjected to electrical stimulation [19].

**Figure 1:**
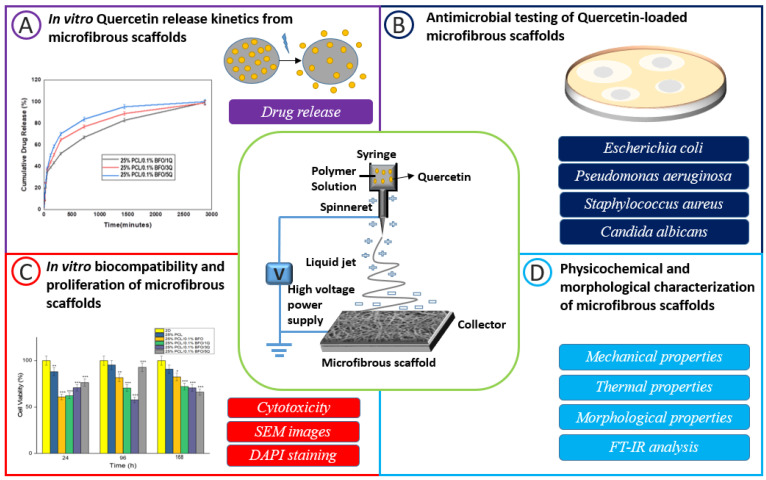
Graphical abstract illustrating the characterization and fabrication of the microfibrous scaffolds. In vitro release of Q from microfibrous scaffolds (**A**). Antimicrobial testing of Q-loaded microfibrous scaffolds (**B**). In vitro biocompatibility and proliferation of microfibrous scaffolds assessed by cytotoxicity test, SEM, and DAPI staining. Significance levels are indicated by * *p* < 0.05, ** *p* < 0.01, and *** *p* < 0.001 (**C**). Physicochemical and morphological characterization of microfibrous scaffolds (**D**).

Electrospun fibrous scaffolds have drawn much attention in the field of tissue engineering because of their customisable porosities, large specific area, and physical resemblance to biological extracellular matrices (ECMs). Recently, it has been reported that functional fibrous scaffolds have been produced that offer different guiding cues, such as topographical [20], electrical [21], or biochemical signals [22,23], for particular cell types to support cell activity and tissue regeneration. Electrospun fibrous scaffolds are responsive to electrical fields and can support the contraction and relaxation of skeletal muscle tissue. They hold great promise in the field of biomedical engineering for regenerating damaged tissues. Electrical signals, which are the primary physical stimuli present in the human body, can modulate cell proliferation and differentiation [24,25], and can also be used to deliver drugs in a controlled manner [26,27]. The fibrous scaffold can be formed by adding metal nanoparticles that induce distinct electrical characteristics in the polymer structure. The electrical characteristics provide plenty of opportunities for cellular processes such as adhesion, cytoskeleton remodelling, differentiation, proliferation, protein secretion, and gene expression; they have also been studied extensively about the numerous cell types, including myoblasts, neurons, fibroblasts, epithelial cells, and muscle cells [28]. In comparison to other methods for creating matrix scaffolds, electrospinning techniques offer particular advantages that meet these essential criteria, such as simplicity in replicating natural ECM’s three-dimensional nano-scaled structures and flexibility in the selection of biocompatible polymers [29]. Moreover, they should be biocompatible to allow cell attachment and proliferation, degradable over time as muscle cells grow into a tissue, and elastic to support contractile action [12]. The electrospinning procedure uses electric charges applied to ejected droplets of a polymeric solution to create nanoscale or microscale fibrous membranes for different synthetic polymers [30]. Due to the enormous effects of skeletal muscle injuries, efforts are being undertaken with great interest using MSCs to develop suitable cell-based and tissue-engineering treatments for bone, tendon, joint, and skeletal muscle problems [31]. The most critical mechanism recommended for MSCs-based treatment is the ability to differentiate into the desired cell type and contribute to the healing of the injured region. PCL was chosen as a synthetic matrix polymer due to its high biodegradability, biocompatibility, and mechanically advantageous tissue-engineering properties [32]. The electrical characteristics of the microfibrous scaffold can be provided by the material BFO, owing to its simplicity of production, affordable and easily accessible ingredients, stability, and lack of toxicity [33]. Q is a flavonoid (polyphenol), and its positive effect has been reported in chronic ageing-related disorders, such as the loss of bone mineral mass (osteoporosis), diabetes, and mesenchymal stem cell differentiation [34]. We have previously shown that the PCL scaffold was combined with BFO by inspecting and comparing electrical, morphological, thermal, and biological properties [35]. In the current study, we examined the skeletal muscle regeneration capability using Q in electrospun PCL/BFO microfibrous scaffolds to design and evaluate their influence on electrically controlled drug release, antimicrobial activity, thermal stability, and microstructural properties (Figure 1). In addition, to investigate the biocompatible properties of PCL/BFO/Q microfibrous scaffolds for skeletal muscle engineering, MSCs were cultured on the microfibrous scaffold to study cell growth and proliferation.

## 2. Materials and Method

### 2.1. Materials

Nitric acid (65%), iron (III) nitrate nonahydrate (Fe(NO_3_)_3_, MW = 403.95 g/mol), bismuth (III) nitrate (Bi_5_O(OH)_9_(NO_3_)_4_, MW = 1.462 g/mol), and dichloromethane (DCM) were acquired from Merck KGaA, Darmstadt, Germany. Ammonia solution (25%, MW = 35.05 g/mol) was purchased from ISOLAB (Wertheim, Germany). PCL (MW = 80,000) was purchased from Sigma Aldrich (Gillingham, UK). Distilled water was supplied by a water distiller (Liston). Q (CAS: 6151-25-3) was obtained from Sigma-Aldrich, Taufkirchen, Germany. Antimicrobial assay: *Staphylococcus* (*S.*) *aureus* ATCC 6538, *Escherichia* (*E.*) *coli* ATCC 8739, *Pseudomonas* (*Ps.*) *aeruginosa* ATCC 9027 and *Candida* (*C.*) *albicans* ATCC 10231 was provided by American Type Culture Collection (ATCC, Manassas, VA, USA).

### 2.2. Preparation of BFO Nanoparticles

Co-precipitation, which involves the simultaneous precipitation of two cations (Bi^3+^ and Fe^3+^), is a valuable technique for creating nano-scaled magnetic particles to obtain precursors. This technique was used in this work to generate BFO nanoparticles. The first step was to dissolve 2.58 g of iron nitrate (Fe (NO_3_)_3_9H_2_O) in 10 mL of distilled water using a magnetic stirrer at 300 rpm for 15 min. Then, 1.86 g of bismuth nitrate (Bi (NO_3_)_3_·5H_2_0) was dissolved in 10 mL of nitric acid for 30 min at the same speed. After the two solutions had completely dissolved, they were combined in a beaker for 15 min to create a uniform mixture. Ammonia solution was added to the mix after it became transparent, to adjust the pH and produce a precipitate. Preparation of the precursor and co-precipitation solution was carried out at room temperature. The resultant precipitate was run through filter paper and rinsed with distilled water to eliminate the harmful effects of the agents. To attain pure crystalline BFO, a heating process was conducted on the powders within an over for a duration of 24 hours at a temperature of 100 °C. Following this, the material was calcined for 3 hours at a temperature of 550 °C.

### 2.3. Preparation of the Solutions

Initially, 25% PCL solutions were prepared as a control group by dissolving 2.5 g PCL in 10 mL DCM. This solution was stirred with magnetic stirrer for one hour at 300 rpm. BFO (0.1%) was independently mixed with 25% PCL solution at the same stirring rpm and ambient temperature, for 30 min. Subsequently, Q was added to PCL/BFO solution at 1, 3, and 5 mg, respectively, and stirred for 1 h. The solutions were categorised according to the content of microfibrous scaffolds, especially the concentration of Q.

### 2.4. Electrospinning

The experimental setup (NS24, Inovenso Co., Istanbul, Turkey) consisted of a single brass needle with a diameter of 1.63 mm, a high-voltage power source attached to the needle, an electrospinning device, and a syringe pump (NE-300, New Era Pump Systems., Farmingdale, NY, USA). The fibers were collected in a grease-proof paper-wrapped metal cylinder. Then, 10 mL suspensions were poured into plastic syringes. The flow rate value was 0.5 mL/h, and the electrospinning parameters were initially a 20–25 kV operational voltage range, and 12 cm needle-to-collector distance. These values were optimised during the electrospinning process.

### 2.5. Characterisation of the Microfibrous Scaffolds

Scanning electron microscopy (SEM, EVO LS 10, ZEISS, München, Germany) was applied to study the surface morphology of the reference and Q-loaded microfibrous scaffolds. Using image analysis software (Olympus AnalySIS, Waltham, MA, USA), 100 fiber diameters were measured to establish the scaffolds’ average diameters.

The microfibrous scaffolds were examined using fourier transform infrared spectroscopy (FTIR, JASCO-4000, Easton, MD, USA) to observe absorption bands and infrared spectra. All spectra were recorded at a resolution of 4 cm^−1^ in the wavenumber range of 4000 to 400 cm^−1^.

The microfibrous scaffolds’ thermal characteristics were assessed using a differential scanning calorimeter (DSC, Shimadzu, Tokyo, Japan). The temperature was changed at 25 °C/min, ranging from 25 °C to 400 °C.

The microfibrous scaffolds’ tensile strength and elongation at break values were measured using the Shimadzu (EZ-X, Tokyo, Japan) tensile testing apparatus. Before the tensile testing, the microfibrous scaffolds were first trimmed to 10 mm wide and 50 mm long. Using a digital micrometer, the microfibrous scaffolds’ thickness values were determined. The mean tensile strength and strain values of the microfibrous scaffolds were determined by conducting triplicate measures of each, with the aim of obtaining precise and reliable data.

Q’s in vitro release characteristics from PCL/BFO microfibrous scaffolds were measured in phosphate-buffered saline (PBS) at 37 °C and pH 7.4. The resultant Q concentration was assessed at various intervals using a UV-Vis spectrophotometer (Shimadzu, Tokyo, Japan). The drug’s linear calibration curve was determined over a wavelength range of 300–400 nm and for four different drug concentrations (0.25, 0.50, 1, 2 mg/mL). The first stage in the drug-release test was to weigh 5 mg of drug-loaded microfibers and place them in eppendorf tubes with 1 mL PBS (pH 7.4). The absorbance measurements were taken after 15 min, 30 min, 1, 2, 3, and 4 h. After each measure, new PBS was utilised. The Q’s release profile was found at 376 nm. Four distinct mathematical models were utilised to evaluate and simulate Q release kinetics from microfibrous scaffolds. These models include the zero-order, first-order, Higuchi, and Hixson–Crowell models, represented by the equations Q_t_ = Q_0_ + K_0_t, log Q_t_ = log Q_0_ − K_1_t/2.303, Q_t_ = KH_t_^0.5^, and Q_0_^1/3^ − Q_t_^1/3^ = log_t_ + log_κ_, respectively. These equations allow us to determine the fraction of drug released at a given time (Q_t_), the initial fraction of drug in solution (Q_0_), and the release constants for zero-order (K_0_), first-order (K_1_), and Higuchi (K_H_) dissolution, as well as the values of n and κ. Through the application of these models, we were able to gain insights into the Q-release behaviour from microfibrous scaffolds.

The electrically triggered drug-release system was adjusted according to our previous study [36]. It was employed to investigate the electrically regulated release of Q from PCL/BFO microfibrous scaffolds. To assess the influence of voltage on drug-release behaviour, PCL/BFO/Q microfibrous scaffolds (5 mg) were weighed and placed in Eppendorf tubes with 1 mL of PBS (pH 7.4). An Ag/Pt electrode was employed to transfer electricity to the PBS in the eppendorf tube. Experiments were carried out at a frequency of 50 Hz with a constant voltage of 10 V, and the constructions were exposed to electricity at specific intervals from 5 s to 60 min. The PBS in the Eppendorf tubes was obtained after applying the electric field. The effect of voltage and current UV spectroscopy (Jenway 7315, Bibby Scientific, Staffordshire, UK) was used to detect the Q-release profile at 376 nm.

Antimicrobial efficacy was assessed using four reference microorganisms: S. *aureus* ATCC 6538, *E. coli* ATCC 8739, *Ps. aeruginosa* ATCC 9027, and *C. albicans* ATCC 10231, and four clinically acquired *S. aureus* strains (from the wound infections collection of the Research Center at the University of Bucharest). Microbial susceptibility was assessed according to CLSI 2019 M100 [37]. Squares of 1 × 1 cm were cut from microfibrous scaffolds and incubated at 36 ± 2 °C for 4 h with 1.5 × 10^8^ CFU/mL microbial suspensions (standard density of 0.5 McFarland). After 4 h of incubation, all microfibrous scaffolds were thoroughly spun on a vortex, and 6 decimal serial dilutions were carried out. The 10 µL from each dilution were plated on solid Mueller Hinton agar, with Sabouraud for microfungi. After 24 h of incubation, plates were read by counting the colonies. Antimicrobial efficacy (AE) was expressed as logarithmic reduction using the formula in Equation (1):

(1) $$L\left(Logarithmic\;reduction\right)=lg\frac{A}{B}$$

*A* is CFU/mL of negative control (initial number of bacteria in the inoculum); *B* is CFU/mL of microfibrous scaffolds (number of bacteria after x time of contact with antimicrobial substance). All tests were performed in triplicate.

The microfibrous scaffolds were sterilised in the 24-well plates overnight using Ultraviolet (UV). The microfibrous scaffolds were cultured in DMEM growth media with 10% FBS, 0.1 mg/mL penicillin/streptomycin, and 5% CO_2_ for an hour, to provide the cells with a microenvironment. Following incubation, the growth media were collected using a micropipette, and the remaining medium was discarded. MSCs were seeded at a density of 10.000 cells per microfibrous scaffold in 24-well plates and allowed to adhere for 24 h. The same number of monolayer cells (2D) were cultured for 7 days in a humidified incubator at 37 °C, 5% CO_2,_ with the cell–microfibrous scaffolds as a control group. A Glentham Life Sciences’ MTT ((3-[4,5-dimethylthiazol-2yl]-2,5-diphenyl-tetrazolium bromide)) cytotoxicity detection kit was employed to test the cytotoxicity of the microfibrous scaffolds at particular time intervals. The absorbance value of the cytotoxicity test at 560 nm wavelength was measured using an Elisa reader (Perkin Elmer, Enspire, Waltham, MA, USA). The test was repeated three times to obtain precise values, and the average values of the results were used as the mean result. The cellular morphology of the cells on the microfibrous scaffolds was observed using a scanning electron microscope (ZEISS, EVO MA 10, Oberkochen, Germany). 

For the purpose of morphological analysis using fluorescence microscopy, MSCs were seeded onto microfibrous scaffolds at a density of 10.000 cells per scaffold in 24-well plates. The microfibrous scaffolds were fixed using 4% formaldehyde for 1 hour, followed by permeabilization with 0.1% Triton X-100 in PBS for 10 minutes, and subsequently rinsed with PBS. Furthermore, 4′,6-diamidino-2-phenylindole (DAPI, Invitrogen) was added at a dilution of 1/5000 to each microfibrous scaffold and kept for 5 minutes at room temperature. The cells on microfibrous scaffolds were then identified using a fluorescent microscope, and images were captured using a Leica DMI 6000B fluorescence microscope (Wetzlar, Germany).

The growing media were removed after 1, 3, and 7 days of incubation, and the microfibrous scaffolds were fixed with 4% glutaraldehyde (Sigma Aldrich). The microfibrous scaffolds were then dehydrated using repeated dilutions of ethanol and air-dried. The dried microfibrous scaffolds were coated with gold and studied under SEM at various magnifications. 

The statistical analysis was performed with GraphPad Prism 9 (San Diego, CA, USA). Data were analysed using the one-way ANOVA test. The level of significance was set to * *p* < 0.05, ** *p* < 0.01, and *** *p* < 0.001.

## 3. Results and Discussions

### 3.1. Morphological Examinations

**Figure 2:**
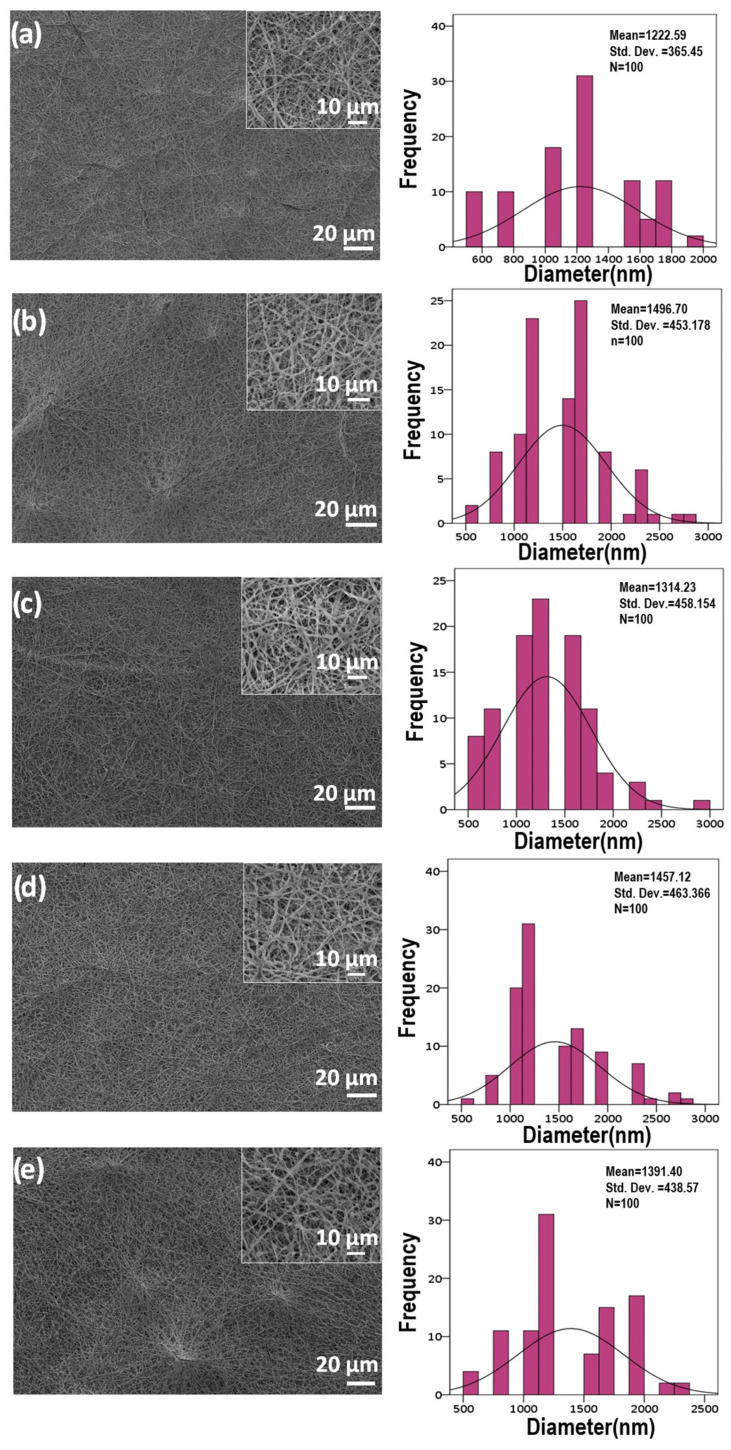
SEM images and fiber diameter distribution of the 25% PCL (**a**), 25% PCL/0.1% BFO (**b**), 25% PCL/0.1% BFO/1Q (**c**), 25% PCL/0.1% BFO/3Q (**d**) and 25% PCL/0.1% BFO/5Q (**e**) microfibrous scaffolds.

An inquiry into the impact of Q on the morphology of microfibrous scaffolds was undertaken, followed by an examination of the scaffold’s structure in the absence of Q. Figure 2 shows SEM images of microfibrous scaffolds and the fiber-orientation histograms for each of the five microfibrous scaffolds, along with the superimposed standard curves. The images of microfibrous scaffolds have no traces of beads or cracks; in parallel with this, a uniform ribbon structure within the fiber formation was observed. The assertion that uniformity fosters optimal conditions for cell proliferation, intercellular communication, and cellular differentiation enjoys significant support in the scientific literature [38]. Using SEM images, all of the microfibrous scaffold’s average fiber diameters were measured to examine quantitatively the impact of Q. As shown in Figure 2a,b, the diameter of the 25% PCL was roughly 1222 ± 365 nm, and when BFO was added, this diameter expanded to around 1496 ± 453 nm. In relation to the amount of Q used in the microfibrous scaffolds, the fiber diameters fluctuated slightly between 1300–1500 nm. The loading of 1 mg of Q (1Q) throughout the microfibrous scaffolds in Figure 2c caused the fiber diameter to drop to the value of 1314 ± 458 nm; conversely, the diameter of 3 mg of Q (3Q) increased to 1457 ± 463 nm in Figure 2d, and the diameter fell negligibly to 1391 ± 438 nm in the event of 5 mg of Q (5Q) being added to the microfibrous scaffold, as in Figure 2e. Given the microenvironment, the averages of all fiber diameters did not differ considerably from one another; however, it should be noted that the incorporation of Q with microfibrous scaffolds has never been associated with increased fiber diameters.

### 3.2. Characterisation of the Microfibrous Scaffolds

**Figure 3:**
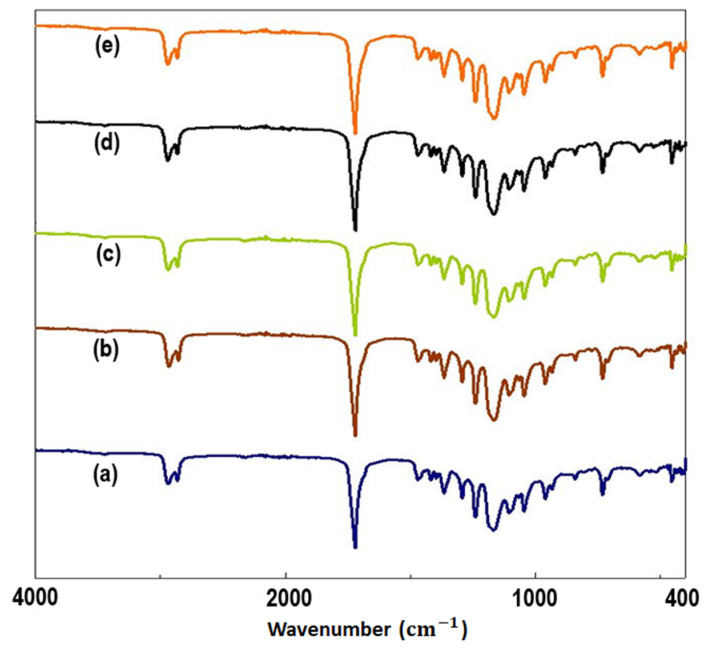
FTIR analysis spectra of 25% PCL (**a**), 25% PCL/0.1% BFO (**b**), 25% PCL/0.1% BFO/1Q (**c**), 25% PCL/0.1% BFO/3Q (**d**) and 25% PCL/0.1% BFO/5Q (**e**) microfibrous scaffolds.

FTIR was applied to reveal the surface functional groups of the microfiber scaffolds, as shown in Figure 3. In Figure 3a, the main absorption peaks of PCL can be seen at 2940 cm^−1^, 1720 cm^−1^, 1238 cm^−1^, and 1164 cm^−1^, corresponding to asymmetric CH_2_, carbonyl, C-O-C, and C-O stretching, respectively [39,40]. In Figure 3b–e, the peaks between 450–525 cm^−1^ can be assigned to the stretching and bending vibrations of Fe-O bonds [35,41]. The peaks observed in the spectrum attributable to the stretching and bending vibrations of Fe-O bonds are indicative of the existence of [FeO_6_] octahedra in perovskites, where the metal ions are linked to these bonds [42]. The vibration of the Bi-O bond occurs at around 1060 cm^−1^ [43]. Figure 3c–e displays the microfibrous scaffolds with the main absorption peaks of PCL. The distinctive peak at 1388 cm^−1^ reveals the OH bending vibration of the phenolic groups of Q, and the wavelength of 1325 cm^−1^, 822 cm^−1^ indicates the C-H group stretching [44]. The OH stretch band of Q at 3400~3420 cm^−1^ [45] was absent or weakened due to intermolecular interactions between Q and PCL. However, the potential involvement of water can be subject to the bending vibration of water, observed at 1642 cm^−1^ or 1630 cm^−1^. These bands can overlap with the C=O vibration that occurs at approximately 1650 cm^−1^, which makes it difficult to distinguish the two signals [46]. The spectra with Q-loaded microfibrous scaffolds demonstrated absorption peaks that were very similar to those of 25% PCL/0.1% BFO. This might be because the microfibrous scaffolds contain only a minor amount of Q, or the hydrogen bonds in the PCL matrices allow Q to be encapsulated there.

### 3.3. Thermal Properties of Microfibrous Scaffolds

**Figure 4:**
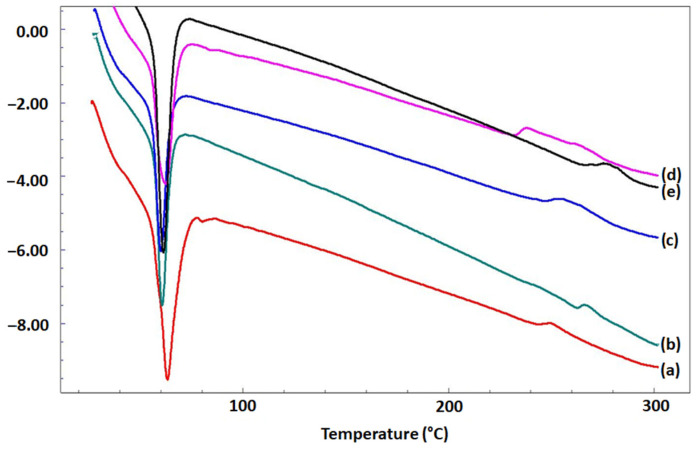
DSC curves of 25% PCL (**a**)**,** 25% PCL/0.1% BFO (**b**), 25% PCL/0.1% BFO/1Q (**c**), 25% PCL/0.1% BFO/3Q (**d**) and 25% PCL/0.1% BFO/5Q (**e**) microfibrous scaffolds.

Figure 4 reveals DSC thermograms of PCL/BFO and the integration of Q in PCL/BFO microfibrous scaffolds. The DSC curves for all microfibrous scaffolds presented two-phase transition peaks. First, the melting temperature (T_m_) value was observed at around 64 °C for microfibrous scaffolds without Q; however, T_m_ values were reduced to 60.2 °C when Q was included in the microfibrous scaffolds. Secondly, the primary decomposition peak was approximately 245 °C for 25% PCL. However, the decomposition peak was slightly altered with the addition of Q. Similar results for the T_m_ peak of PCL have been reported in the literature [47]. Compared with our conclusions, previous studies showed that the decomposition of Q was initiated at an earlier stage, approximately around 230 °C [46]. Proceeding from this, it can be inferred that the thermal behaviour of Q was improved by emulating the thermal behaviour of PCL. Accounting for the similarity in DSC results between three distinct types of materials, synthetic, multiferroic, and flavonoid, it can be understood that Q appears miscible with PCL and BFO. The production of an appropriate PCL-based fibrous structure by electrospinning, as well as the production of biocomposites with suitable mechanical properties and drug-release profiles, should benefit significantly from these miscibility properties.

### 3.4. The Effect of Q on Mechanical Properties

Investigating the mechanical characteristics of these scaffolds is crucial to ensure their suitability for tissue-engineering applications. This is particularly important for microfibrous scaffolds designed for skeletal muscle tissue engineering, as they need to have adequate mechanical properties to promote muscle cell growth, increase cell elongation, and enhance muscle cell fusion. Table 1 summarises the mechanical characteristics of the microfibrous scaffolds calculated from the stress–strain curves. Several concentrations of Q were combined with 25% PCL/0.1% BFO scaffolds to determine the impact on mechanical strength. The tensile strength and strain at break were increased when the concentration of Q increased among the Q-loaded microfibrous scaffolds. The highest tensile strength (7.67 ± 0.52 MPa) was observed with 25% PCL, and the highest strain at break (27.74 ± 7.86%) with 25%PCL/0.1% BFO. The addition of Q into Q-loaded microfibrous scaffolds resulted in significant improvement in their mechanical properties, specifically in the areas of tensile strength and elongation. The mechanical properties of PCL microfibrous scaffolds may have been improved if the Q concentration were greater than 10 mg. However, the values we obtained were satisfactory to meet the criteria for skeletal muscle regeneration. Additionally, it should be noted that given the representative tensile strength and elongation of human muscle tissue of 0.1 MPa and 20%, respectively [48], the mechanical properties of all Q-loaded microfibrous scaffolds may be within the clinically relevant range for the regeneration of skeletal muscle. The elasticity of microfibrous scaffolds represents a promising approach to enhance muscle regeneration, as it may counteract the detrimental effects of inappropriate mechanical cues associated with injured or diseased muscle tissue. The resting elasticity in healthy muscle tissue is estimated to be 12 kPa. However, various factors, including aging, injury, and disease, can result in tissue stiffening, leading to an elastic modulus exceeding 18 kPa [49]. Recent studies have demonstrated the potential role of elasticity in the regulation of mesenchymal stem cell (MSC) differentiation, where extracellular matrices that mimic the elasticity of striated muscle tissue, ranging from 8 to 17 kPa, have been shown to promote myogenic differentiation [50]. The results indicate that the elastic modulus of the microfibrous scaffold decreased with the addition of Q, but still remained higher than the values reported in the literature for skeletal muscle tissue. Therefore, the Q-added scaffold may be a promising candidate for skeletal tissue engineering, as it provides adequate mechanical support for the newly formed tissue.

### 3.5. In Vitro Drug Release

**Figure 5:**
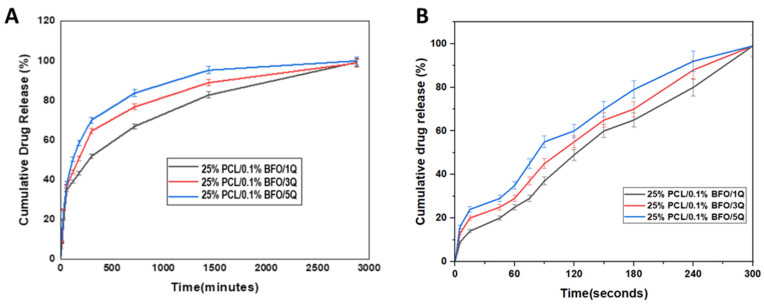
Cumulative drug-release graph of Q from the drug-loaded microfibrous scaffolds (**A**), and under electric stimulus with 50 Hz frequency (**B**). Electrically triggered Q release from microfibrous scaffolds.

The results of an in vitro drug-release investigation, shown in Figure 5A, were analysed to examine the drug release profile of Q-loaded microfibrous scaffolds. The calibration curve and absorbance graph at 376 nm were reported in our previous study [36]. During the first 1 h of incubation, 25% PCL/0.1% BFO/1Q, 25% PCL/0.1% BFO/3Q, and 25% PCL/0.1% BFO/5Q exhibited initial burst release of around 34.80%, 36.80%, and 38.15%. The initial burst release of the drug was likely caused by the entrapped drug leaching out from the surface of the microfibrous scaffolds [51]. After 24 h immersion, 82.75, 89, and 95.31% of Q were released from 25% PCL/0.1% BFO/1Q, 25% PCL/0.1% BFO/3Q, and 25% PCL/0.1% BFO/5Q, respectively. A steady and prolonged drug release was observed up to 48 h. Sustained drug release from Q-loaded microfibrous scaffolds might be due to the presence of PCL in the scaffolds, which decreases PBS penetration into fibers [52]. The drug-holding capabilities of microfibrous scaffolds may be controlled by microfiber thickness; however, the diameters of all Q-loaded microfibrous scaffolds were very similar to each other. An observed trend in the release kinetics of Q from microfibrous scaffolds indicated that its release capacity was positively correlated with increasing concentrations of Q. Figure 5B depicts Q electrically released from microfibrous scaffolds. We examined the impact of the applied electric field because, in this situation, the usage of BFO could impart electrically induced delivery. The effect of the electric field on Q release from the 25% PCL/0.1% BFO microfibrous scaffolds was assessed at 50 Hz frequency. When the release behaviour was investigated under an electric field with a frequency of 50 Hz, the 25% PCL/0.1% BFO/5Q microfibrous scaffold had the highest release percentage after 180 s of stimulation, with a cumulative release value of approximately 79%. The 25% PCL/0.1% BFO/1Q microfibrous scaffold showed the lowest proportion of Q release, which gradually increased until 300 s. The cumulative release for all scaffolds was similar and proportional to the content of Q during the whole period of time. All obtained PCL/0.1% BFO/Q microfibrous scaffolds reached 100% cumulative release after 300 s of electric stimulation. It was shown that 100% of the drug was released after 5 min of stimulation under an electric field with a frequency of 50 Hz, but conversely, all microfibrous scaffolds were released in 2880 min. This release speed is roughly 576 times faster than the conventional drug-release method without any electrical stimulation. It was revealed that the application of an electric field induced rapid release of the drug. The expedited delivery of therapeutic agents to the injury site through rapid drug release may provide improved drug efficiency [53]. This attribute is especially crucial in scenarios where a limited time window exists to facilitate regeneration, as is the case with acute injuries. The mechanism for faster release in the presence of an electric field can be explained by the concept of electrostatic interaction. When an electric field is applied to a charged polymeric system, the charged molecules within the system start to move towards the oppositely charged electrode. This movement generates a repulsive force between the molecules, which in turn causes the polymer chains to stretch and expand. As a result of this expansion, the porosity of the polymer matrix increases, allowing the release of the encapsulated drug to occur at a faster rate [54]. The study findings suggest that 25% PCL/0.1% BFO/5Q scaffolds can be employed as controlled drug-delivery systems with electric triggering capability. The ability to change the electrical field may facilitate the timed release of Q from 25% PCL/0.1% BFO/5Q microfibrous scaffolds at a desired rate, providing more precise and effective therapy for the treatment of various diseases and injuries. The delivery can be tailored to the patient’s demands by altering the properties of the applied current. In this instance, a simple electric field can be used to fine-tune the delivery, and this can be achieved simply by altering the frequency. Moreover, electrically triggered drug release can also overcome the limitations of conventional drug-delivery systems, which may include low drug efficacy and potential side effects [55]. Although it has been shown that scaffolds can be utilised with the ability to be triggered electrically, suggesting that they can find application in personalised medicine, further optimisations must undoubtedly be carried out by altering the electric field properties. In addition to using these biomaterials for prolonged drug delivery, properly designed materials may also be utilised to support the sustained release of functional cells into the surrounding tissue while protecting transplanted cells from the hard tissue. Moreover, it is highly desirable that the materials biodegrade with kinetics that mimics skeletal muscle’s natural healing process, as intensely rapid degradation could result in open areas filled with scar tissue; however, prolonged degradation could require invasive surgical removal to prevent a protracted immune response [56,57].

**Figure 6:**
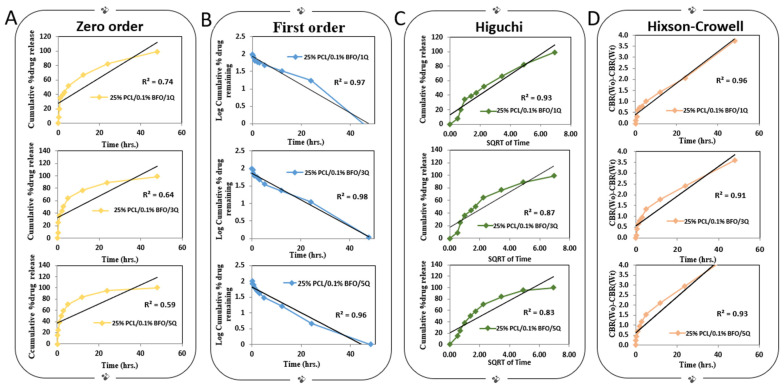
Drug-release studies of Q-loaded microfibrous scaffolds, zero-order drug-release kinetics (**A**), first-order drug-release kinetics (**B**), Higuchi drug-release kinetics (**C**), Hixson–Crowell drug-release kinetics (**D**).

The kinetics of Q release from microfibrous scaffolds incubated under dynamic conditions in PBS (pH 7.4) were examined using various release models including zero order, first order, Higuchi, and Hixson–Crowell (Figure 6). The efficacy of each model was evaluated by determining its correlation coefficient (R^2^), and the model that produced the highest correlation coefficient was deemed the most suitable kinetic model for the release of Q from the microfibrous scaffolds. The first-order kinetic model explains more than 96% of the variance in the experimental data, which suggests that the model is highly accurate in predicting the kinetics of Q release. The first-order kinetic model assumes that the rate of drug release is proportional to the concentration of the drug remaining in the matrix. This model is often used to describe the release of drugs from polymeric matrices or other delivery systems. Among the several viable drug kinetic models, the Hixson–Crowell model represents another appropriate choice. This model is based on the underlying assumption that the rate of drug release is predominantly governed by the rate of dissolution rather than diffusion, which can occur through the polymeric matrix. The R^2^ value of the Hixson–Crowell model was found to be at least 91%, indicating a strong correlation between the experimental data and the model predictions. Moreover, microfibrous scaffolds containing a loading dose of 25% PLC/0.1% BFO/1Q exhibited an even higher R^2^ value of around 96%, further supporting the efficacy of the Hixson–Crowell model in accurately describing the drug-release kinetics of such systems. The zero-order model may not accurately capture the complexity of the drug-release kinetics, leading to a poor fit with the experimental data when compared with the other kinetic models. The selection of the appropriate kinetic model for drug release depends on the specific application and the desired release profile. In the case of skeletal regeneration, it is important to achieve sustained release of the drug over a prolonged period of time to support bone growth and repair [58]. Both the Hixson–Crowell and first-order kinetic models may provide sustained drug release, although their underlying mechanisms differ.

### 3.6. Antibacterial Activity

**Figure 7:**
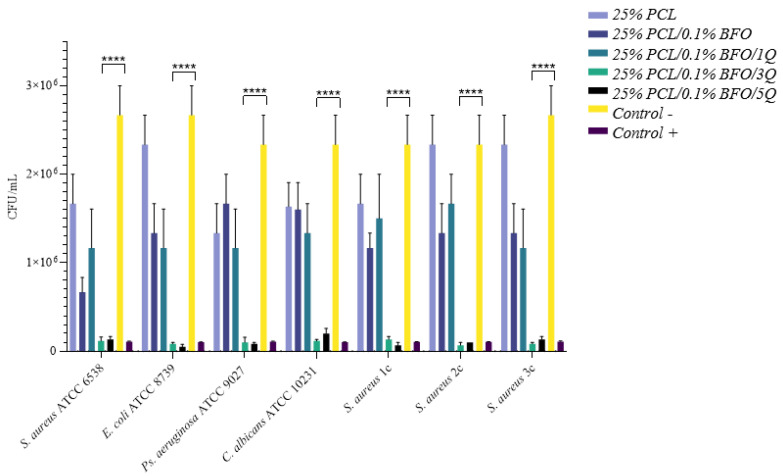
Antimicrobial susceptibility of microfibrous scaffolds, as CFU/mL reduction. Positive control = 5 mg/mL of Q solution. Negative control = untreated bacterial suspensions. **** *p* value < 0.002.

**Figure 8:**
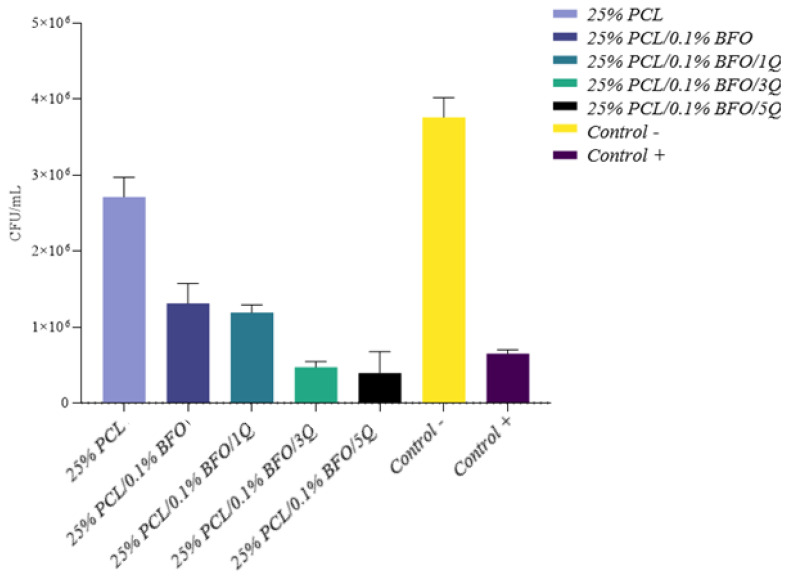
Depiction of summary of antimicrobial efficacy expressed as CFU/mL reduction for microfibrous scaffolds, as CFU/mL reduction. Positive control = 5 mg/mL Q solution.

**Figure 9:**
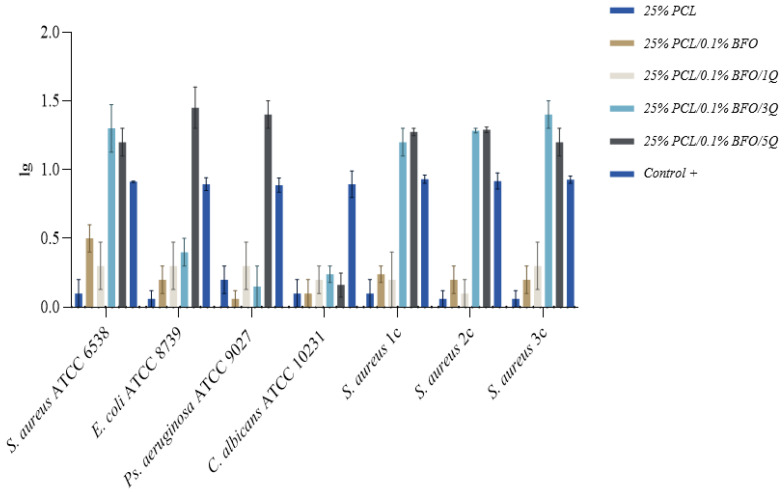
Antimicrobial efficacy expressed as lg for microfibrous scaffolds, as CFU/mL reduction. Positive control = 5 mg/mL Q solution.

A successful scaffold design incorporates potent antimicrobial substances capable of reducing or preventing microbial adhesion and growth, to achieve total inhibition and proper muscle regeneration polymers. Synthetic polymers such as PCL do not possess inherent antibacterial properties [59]. Although passive resistance against bacteria can be provided by making the scaffold hydrophilic, several studies recommend the embedding of a potent antimicrobial [60,61,62] to achieve the desired effect. Q is a bioflavonoid with a wide range of physiological and pharmacological activities relevant to human health [59,63], with reportedly good antimicrobial activity [64,65,66] effectively reducing the formation of biofilms through inhibiting the expression of related genes [66]. Results obtained in this study indicate high antimicrobial efficacy for 25%/PLC/0.1% BFO/5Q, as in Figure 7, with a median reduction of over 50% across the tested strains. Next in the efficacy ranking follows 25% PLC/0.1%BFO/3Q, both microfibrous scaffolds representing good candidates for skeletal muscle tissue applications because they able to promote a cleaner and more conducive environment for tissue growth and regeneration, leading to better outcomes. A depiction of the summary of the antimicrobial efficacy (AE) (Figure 8) of 25% PLC microfibrous scaffolds loaded with Q and 0.1% BFO distinctly represented the overall effect of 25% PLC/0.1% BFO/3Q, and 25% PLC/0.1% BFO/5Q on tested strains, similar to the 5 mg/mL Q solution tested as a positive control, and for 25% PLC a diminished AE was observed, with values tending to be identical to the negative control. 25% PLC/0.1% BFO/5Q presented the highest efficiency against all tested strains, standardised as well as clinically acquired (from infected wounds), with logarithmic reductions (lg) (Figure 9) ranging from 0.3 to 1.6. The reduction levels over 1 lg signify over 90% efficacy in microbial reduction. For 25% PLC/0.1% BFO/5Q, a 0.3 lg reduction was obtained for the *Candida albicans* strain. The next highest level of effect was 25% PLC/0.1% BFO/3Q with lg ranging from 0.3 to 1.6. Reductions of 0.3 to 0.6 lg were observed for *E. coli*, *Ps. Aeruginosa*, and *C. albicans*. For *S. aureus* strains, reductions of 1 to 1.6 lg were obtained. Both microfibrous scaffolds presented outcomes similar to the positive control (control+) represented by a Q solution of 5 mg/mL. We observed that the microbicidal effect of microfibrous scaffolds increased significantly with an increment of Q concentration. As we now know, excessive use of antibiotics leads to the development of antibiotic-resistant microorganisms, which creates further significant problems; therefore, using biomolecules that are unlikely to cause resistance is a beneficial solution. One of the main advantages of incorporating Q into the microfibrous scaffold is its broad spectrum of biological activity [60]. However, because its chemical stability depends on pH, temperature, light and the oxidative environment, its bioavailability is unstable, and a suitable delivery system must be considered.

### 3.7. Biocompatibility Properties of the Microfibrous Scaffolds

**Figure 10:**
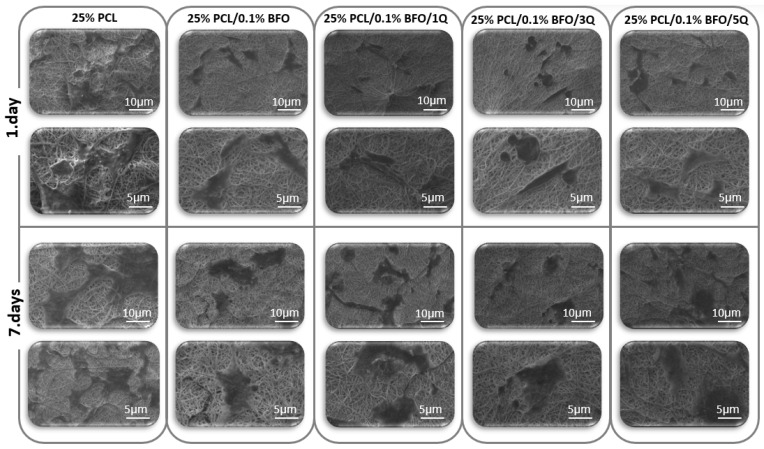
SEM images of the cultured MSCs’ adhesion to each microfibrous scaffold after 1 and 7 days of incubation.

**Figure 11:**
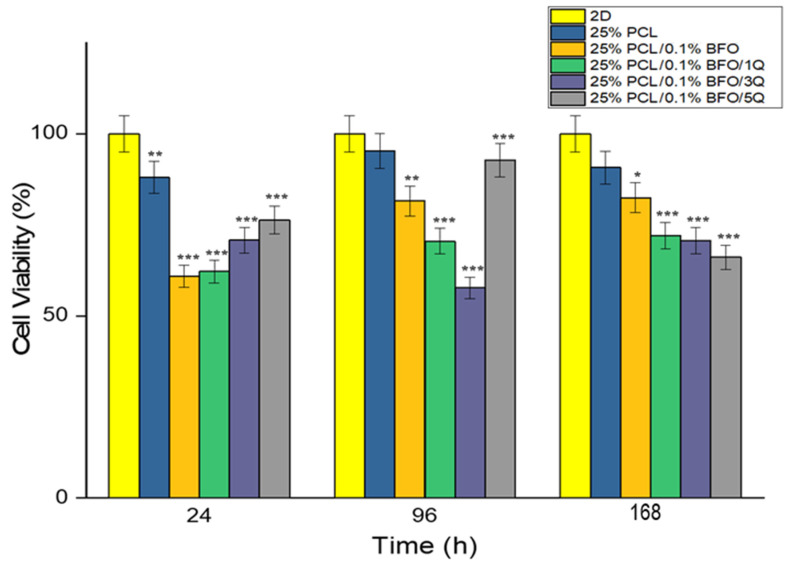
MSC viability of 6 microfibrous scaffolds at 24, 96 and 168 h. Statistical significance was determined using one-way ANOVA Tukey-Kramer multiple comparisons test, with comparison to 2D. * *p* < 0.05, ** *p* < 0.01, and *** *p* < 0.001 indicate significance levels.

**Figure 12:**
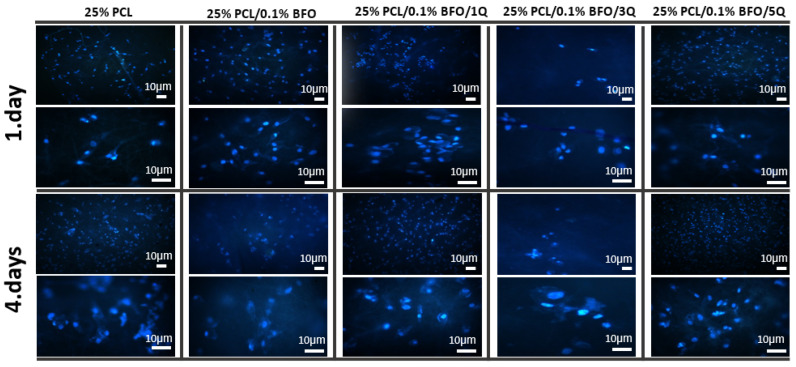
Fluorescence images of MSC viability on the microfibrous scaffolds after 1 and 4 days of the culture period.

According to Yamaoka et al., the designed scaffold should provide the target cells with the necessary space to function and should support their activities. To create an optimal scaffold, all the target cells’ characteristics should be taken into account, as the microfibrous scaffolds are designed to mimic the living tissue in their mechanical properties, which in turn plays a key role in cell adaptation and proliferation and could be used in therapeutic protocols [67]. In this study, the viability of MSCs was tested by culturing them in different scaffolds for 7 days, and samples were later tested by MTT assay. The MTT assay results showed that MSCs cells could attach and proliferate on each scaffold. As shown in Figure 10, there were no significant morphological differences between the cultured MSCs even with the addition of BFO and Q. However, the cell distribution on the 25% PCL microfibrous scaffold seems more uniform and has higher cell connectivity. On the other hand, if we consider the morphological differences between the microfibrous scaffolds by incubation time, we can see that the cell distribution and visibility were higher on the 7th day. Cell proliferation increased, and cells’ distribution on scaffolds appeared more widespread and uniform. Figure 11 displays the cell viability test conducted in vitro over 24, 96, and 168 h. As can be seen, the cell viability was highest in the first 24 h, compared with MSCs’ viability at 96 and 168 h. In addition, the cell viability of the 25% PCL microfibrous scaffolds resembled the 2D sample’s cell viability pattern, and the cell viability of 25% PCL microfibrous scaffolds was 88%. This can be attributed to the MSCs’ ease of adaptability to the new environment and the existence of sufficient amounts of fresh nutrients. Additionally, the cell viability measurements for 25% PCL/0.1% BFO/1Q, and 25% PCL/0.1% BFO/5Q were 62.1% and 76.3%, respectively, at 24 h. In the following 72 h, we observed an increased cell growth compared to MSCs’ viability at 24 h, except for the 25% PCL/0.1% BFO/3Q scaffold, where the cell viability decreased from 70.8% to 57.6%. Day 4 cell viability results show that 25% PCL (95.3%) and 25% PCL/0.1% BFO/5Q (92.8%) scaffolds have the highest MSCs among all the microfibrous scaffolds. For the other two microfibrous scaffolds, 25%PCL/0.1%BFO and 25%PCL/0.1%BFO/1Q, cell viability at day 4 was recorded as 81.5% and 70.5%, respectively. These values demonstrate how the antioxidant and free radical-scavenging characteristics of Q influenced cell viability [68,69]. Although there was no vast difference between cell viability at 72 and 168 h, cell viability decreased slightly at 168 h, which could be a result of the degradation rate of the microfibrous scaffolds or of the proliferation rate. Therefore, it is necessary to conduct additional research on the association between degradation and cell viability in order to achieve the best results and optimised conditions. Nevertheless, the cell viability contributed to proliferation and attachment on most of the microfibrous scaffolds, which is regarded as attributable to the non-toxic nature of Q [68]. MSCs’ adhesion on the microfibrous scaffolds was visualised using fluorescence microscopy, as shown in Figure 12. This revealed that the morphological differences between microfibrous scaffolds are proportional to the incubation time. The highest adherent cell density of MSCs was observed on the surface of 25% PCL/0.1% BFO/5Q, which might have resulted from the unique bioflavonoid Q in the microfibrous scaffold [70]. The addition of BFO and Q was not found to change the cells’ adhesion behaviour on the microfibrous scaffolds in any significant way. However, adding BFO and Q provided a more suitable environment for MSCs to spread on the surface of the microfibrous scaffolds, and ensured higher viability than the 25% PCL. The microfibrous scaffold that showed the lowest increase in cell adhesion and viability even after the 4th day, compared with the other microfibrous scaffolds, was 3 mg of Q. The MSCs were able to spread and proliferate after 4 days, and their shape on each microfibrous scaffold became more extensive and more visible than the structure of the cells on the 1st day.

## 4. Conclusions

In this study, 25%PCL/0.1% BFO/Q microfibrous scaffold was produced by electrospinning, included smooth, bead-free, hydrophilic features, and had good mechanical properties. The addition of BFO can induce electric triggering capacity, thus, electrical stimulation can be applied to the microfibrous scaffolds in order to personalise the delivery rate by changing the parameters of the applied current. According to the obtained results, the release of the drug from 25%PCL/0.1% BFO happened in just 5 min when using an electric field with a frequency of 50 Hz, while thousands of minutes were needed for 100% release of Q without the application of an electric field. The microfibrous scaffolds were seeded in MSCs for 1, 4, and 7 days to determine in vitro biocompatibility. Cells were found to have attached to the Q-loaded microfibrous scaffolds, and their proliferation was carefully investigated through fluorescence and SEM images. 5Q-loaded microfibrous scaffolds showed the best cell activity among the Q-loaded scaffolds, until day 7. Regarding the mechanical experiment, the 25% PLC/0.1% BFO/5Q microfibrous scaffold provided the most effective results by revealing the highest tensile strength and strain amongst the Q-loaded microfibrous scaffolds. Q-loaded microfibrous scaffolds also presented significant antimicrobial efficacy, especially 25% PLC/0.1% BFO/5Q. Results indicate a > 90% microbial reduction capacity correlated with an lg ranging from 0.3 to 1.6, with the highest reduction observed with *S. aureus* strains. Taking into consideration the increased incidence of skin infections associated with *S. aureus* strains, in view of these results, 25% PLC/0.1% BFO/5Q was chosen as a potential microfibrous scaffold for skeletal muscle tissue engineering. The properties of electric-stimuli responsive microfibrous scaffolds can be utilised for the purpose of releasing therapeutic molecules in various biological environments. The ability to achieve precise and controlled drug delivery may potentially enable novel treatments for skeletal muscle tissue. Further study will focus on exploiting this product‘s potential for usage in a variety of animal models.

## Figures and Tables

**Table 1:** The tensile strength (MPa), strain at break (%), and elastic modulus (MPa) values of each microfibrous scaffold.

Fibers	Tensile Strength (MPa)	Strain at Break (%)	Elastic Modulus (MPa)
25% PCL	7.67 ± 0.52	25.42 ± 8.76	35.19 ± 4.24
25% PCL/0.1% BFO	7.09 ± 0.42	27.74 ± 7.86	28.45 ± 5.53
25% PCL/0.1% BFO/1Q	2.11 ± 0.51	8.57 ± 1.65	25.48 ± 7.04
25% PCL/0.1% BFO/3Q	4.24 ± 0.84	17.92 ± 3.90	25.43 ± 7.06
25% PCL/0.1% BFO/5Q	4.52 ± 0.64	20.80 ± 3.49	23.79 ± 5.18

## Data Availability

Upon request, data can be provided.

## References

[1] Zhu M., Li W., Dong X., Yuan X., Midgley A.C., Chang H., Wang Y., Wang H., Wang K., Ma P.X. (2019). In vivo engineered extracellular matrix scaffolds with instructive niches for oriented tissue regeneration. Nat. Commun..

[2] Qazi T.H., Mooney D.J., Pumberger M., Geißler S., Duda G.N. (2015). Biomaterials based strategies for skeletal muscle tissue engineering: Existing technologies and future trends. Biomaterials.

[3] Hurme T., Kalimo H., Lehto M., Järvinen M. (1991). Healing of skeletal muscle injury: An ultrastructural and immunohistochemical study. Med. Sci. Sports Exerc..

[4] Campion D.R. (1984). The muscle satellite cell: A review. Int. Rev. Cytol..

[5] Papy-Garcia D., Barbosa I., Duchesnay A., Saadi S., Caruelle J.P., Barritault D., Martelly I. (2002). Glycosaminoglycan mimetics (RGTA) modulate adult skeletal muscle satellite cell proliferation in vitro. J. Biomed. Mater. Res. Off. J. Soc. Biomater. Jpn. Soc. Biomater. Aust. Soc. Biomater. Korean Soc. Biomater..

[6] Wozniak A.C., Pilipowicz O., Yablonka-Reuveni Z., Greenway S., Craven S., Scott E., Anderson J.E. (2003). C-Met Expression and Mechanical Activation of Satellite Cells on Cultured Muscle Fibers. J. Histochem. Cytochem..

[7] Saxena A.K., Marler J., Benvenuto M., Willital G.H., Vacanti J.P. (1999). Skeletal Muscle Tissue Engineering Using Isolated Myoblasts on Synthetic Biodegradable Polymers: Preliminary Studies. Tissue Eng..

[8] Payumo F.C., Kim H.D., Sherling M.A., Smith L.P., Powell C., Wang X., Keeping H.S., Valentini R.F., Vandenburgh H.H. (2002). Tissue engineering skeletal muscle for orthopaedic applications. Clin. Orthop. Relat. Res..

[9] Powell C.A., Smiley B.L., Mills J., VanDenburgh H.H. (2002). Mechanical stimulation improves tissue-engineered human skeletal muscle. Am. J. Physiol. Physiol..

[10] Negroni E., Butler-Browne G., Mouly V. (2006). Myogenic stem cells: Regeneration and cell therapy in human skeletal muscle. Pathol. Biol..

[11] Sampaolesi M., Torrente Y., Innocenzi A., Tonlorenzi R., D’Antona G., Pellegrino M.A., Barresi R., Bresolin N., De Angelis M.G.C., Campbell K.P. (2003). Cell Therapy of α-Sarcoglycan Null Dystrophic Mice Through Intra-Arterial Delivery of Mesoangioblasts. Science.

[12] Choi J.S., Lee S.J., Christ G.J., Atala A., Yoo J.J. (2008). The influence of electrospun aligned poly (epsilon-caprolactone)/collagen nanofiber meshes on the formation of self-aligned skeletal muscle myotubes. Biomaterials.

[13] Lam M.T., Sim S., Zhu X., Takayama S. (2006). The effect of continuous wavy micropatterns on silicone substrates on the alignment of skeletal muscle myoblasts and myotubes. Biomaterials.

[14] Huang Y.-C., Dennis R.G., Larkin L.M., Baar K. (2005). Rapid formation of functional muscle in vitro using fibrin gels. J. Appl. Physiol..

[15] Riboldi S.A., Sampaolesi M., Neuenschwander P., Cossu G., Mantero S. (2005). Electrospun degradable polyesterurethane membranes: Potential scaffolds for skeletal muscle tissue engineering. Biomaterials.

[16] Bian W., Bursac N. (2008). Tissue engineering of functional skeletal muscle: Challenges and recent advances. IEEE Eng. Med. Biol. Mag..

[17] Mueller C., Trujillo-Miranda M., Maier M., Heath D.E., O’Connor A.J., Salehi S. (2020). Effects of External Stimulators on Engineered Skeletal Muscle Tissue Maturation. Adv. Mater. Interfaces.

[18] Langelaan M.L.P., Boonen K.J.M., Rosaria-Chak K.Y., van der Schaft D.W.J., Post M.J., Baaijens F.P.T. (2010). Advanced maturation by electrical stimulation: Differences in response between C2C12 and primary muscle progenitor cells. J. Tissue Eng. Regen. Med..

[19] Ebrahimi M., Ostrovidov S., Salehi S., Kim S.B., Bae H., Khademhosseini A. (2018). Enhanced skeletal muscle formation on microfluidic spun gelatin methacryloyl (GelMA) fibres using surface patterning and agrin treatment. J. Tissue Eng. Regen. Med..

[20] Jiang X., Cao H.Q., Shi L.Y., Ng S.Y., Stanton L.W., Chew S.Y. (2012). Nanofiber topography and sustained biochemical signaling enhance human mesenchymal stem cell neural commitment. Acta Biomater..

[21] Shao S., Zhou S., Li L., Li J., Luo C., Wang J., Li X., Weng J. (2011). Osteoblast function on electrically conductive electrospun PLA/MWCNTs nanofibers. Biomaterials.

[22] Montero R.B., Vial X., Nguyen D.T., Farhand S., Reardon M., Pham S.M., Tsechpenakis G., Andreopoulos F.M. (2012). bFGF-containing electrospun gelatin scaffolds with controlled nano-architectural features for directed angiogenesis. Acta Biomater..

[23] Lam H.J., Patel S., Wang A., Chu J., Li S. (2010). In Vitro Regulation of Neural Differentiation and Axon Growth by Growth Factors and Bioactive Nanofibers. Tissue Eng. Part A.

[24] Hardy J.G., Lee J.Y., Schmidt C.E. (2013). Biomimetic conducting polymer-based tissue scaffolds. Curr. Opin. Biotechnol..

[25] Ning C., Zhou Z., Tan G., Zhu Y., Mao C. (2018). Electroactive polymers for tissue regeneration: Developments and perspectives. Prog. Polym. Sci..

[26] Kathryn F.A.C., John G.H. (2017). Gene delivery with organic electronic biomaterials. Curr. Pharm. Des..

[27] Svirskis D., Travas-Sejdic J., Rodgers A., Garg S. (2010). Electrochemically controlled drug delivery based on intrinsically conducting polymers. J. Control. Release.

[28] Mushtaq F., Torlakcik H., Vallmajo-Martin Q., Siringil E.C., Zhang J., Röhrig C., Shen Y., Yu Y., Chen X.-Z., Müller R. (2019). Magnetoelectric 3D scaffolds for enhanced bone cell proliferation. Appl. Mater. Today.

[29] Li W.J., Laurencin C.T., Caterson E.J., Tuan R.S., Ko F.K. (2002). Electrospun nanofibrous structure: A novel scaffold for tissue engineering. J. Biomed. Mater. Res. Off. J. Soc. Biomater. Jpn. Soc. Biomater. Aust. Soc. Biomater. Korean Soc. Biomater..

[30] Li D., Wang Y., Xia Y. (2004). Electrospinning Nanofibers as Uniaxially Aligned Arrays and Layer-by-Layer Stacked Films. Adv. Mater..

[31] Torres-Torrillas M., Rubio M., Damia E., Cuervo B., Del Romero A., Peláez P., Chicharro D., Miguel L., Sopena J.J. (2019). Adipose-derived mesenchymal stem cells: A promising tool in the treatment of musculoskeletal diseases. Int. J. Mol. Sci..

[32] Alvarez-Perez M.A., Guarino V., Cirillo V., Ambrosio L. (2010). Influence of Gelatin Cues in PCL Electrospun Membranes on Nerve Outgrowth. Biomacromolecules.

[33] Spaldin N.A. (2020). Multiferroics beyond electric-field control of magnetism. Proc. R. Soc. A.

[34] Hatzistergos K., Quevedo H., Oskouei B.N., Hu Q., Feigenbaum G.S., Margitich I.S., Mazhari R., Boyle A., Zambrano J.P., Rodriguez J.E. (2010). Bone Marrow Mesenchymal Stem Cells Stimulate Cardiac Stem Cell Proliferation and Differentiation. Circ. Res..

[35] Ulag S., Kalkandelen C., Bedir T., Erdemir G., Kuruca S.E., Dumludag F., Ustundag C.B., Rayaman E., Ekren N., Kilic B. (2020). Fabrication of three-dimensional PCL/BiFeO3 scaffolds for biomedical applications. Mater. Sci. Eng. B.

[36] Croitoru A.M., Karaçelebi Y., Saatcioglu E., Altan E., Ulag S., Aydoğan H.K., Sahin A., Motelica L., Oprea O., Tihauan B.M. (2021). Electrically triggered drug delivery from novel electrospun poly (lactic acid)/graphene oxide/quercetin fibrous scaffolds for wound dressing applications. Pharmaceutics.

[37] Clinical and Laboratory Standards Institute (2019). M100 Performance Standards for Antimicrobial Susceptibility Testing: A CLSI Supplement for Global Application.

[38] Liang D., Hsiao B.S., Chu B. (2007). Functional electrospun nanofibrous scaffolds for biomedical applications. Adv. Drug Deliv. Rev..

[39] Ege Z.R., Akan A., Oktar F.N., Lin C.C., Kuruca D.S., Karademir B., Sahin Y.M., Erdemir G., Gunduz O. (2019). Indocyanine green based fluorescent polymeric nanoprobes for *in vitro* imaging. J. Biomed. Mater. Res. Part B Appl. Biomater..

[40] Abdillah M.N., Triyono D. (2019). Structural properties of bismuth ferrite synthesized by sol-gel method with variation of calcination temperature. J. Physics Conf. Ser..

[41] Aliakbari A., Seifi M., Mirzaee S., Hekmatara H. (2015). Influence of different synthesis conditions on properties of oleic acid-coated-FeO nanoparticles. Mater. Sci.-Pol..

[42] Chen C., Cheng J., Yu S., Che L., Meng Z. (2006). Hydrothermal synthesis of perovskite bismuth ferrite crystallites. J. Cryst. Growth.

[43] Troyanchuk I.O., Chobot A.N., Mantytskaya O.S., Tereshko N.V. (2010). Magnetic properties of Bi (Fe_1−x_M_x_)O_3_(M = Mn, Ti). Inorg. Mater..

[44] Catauro M., Papale F., Bollino F., Piccolella S., Marciano S., Nocera P., Pacifico S. (2015). Silica/quercetin sol–gel hybrids as antioxidant dental implant materials. Sci. Technol. Adv. Mater..

[45] Bruno F.F., Trotta A., Fossey S., Nagarajan S., Nagarajan R., Samuelson L.A., Kumar J. (2010). Enzymatic Synthesis and Characterization of PolyQuercetin. J. Macromol. Sci. Part A.

[46] Tsioptsias C., Tsivintzelis I. (2022). On the Thermodynamic Thermal Properties of Quercetin and Similar Pharmaceuticals. Molecules.

[47] Ayran M., Dirican A.Y., Saatcioglu E., Ulag S., Sahin A., Aksu B., Croitoru A.-M., Ficai D., Gunduz O., Ficai A. (2022). 3D-Printed PCL Scaffolds Combined with Juglone for Skin Tissue Engineering. Bioengineering.

[48] Mirfakhrai T., Madden J.D.W., Baughman R.H. (2007). Polymer artificial muscles. Mater. Today.

[49] Cosgrove B.D., Gilbert P.M., Porpiglia E., Mourkioti F., Lee S.P., Corbel S.Y., Llewellyn M.E., Delp S.L., Blau H.M. (2014). Rejuvenation of the muscle stem cell population restores strength to injured aged muscles. Nat. Med..

[50] Engler A.J., Sen S., Sweeney H.L., Discher D.E. (2006). Matrix Elasticity Directs Stem Cell Lineage Specification. Cell.

[51] Karuppannan S.K., Ramalingam R., Khalith S.M., Musthafa S.A., Dowlath M.J.H., Munuswamy-Ramanujam G., Arunachalam K.D. (2021). Copper oxide nanoparticles infused electrospun polycaprolactone/gelatin scaffold as an antibacterial wound dressing. Mater. Lett..

[52] Baghersad S., Hajir Bahrami S., Mohammadi M.R., Mojtahedi M.R.M., Milan P.B. (2018). Development of biodegradable electrospun gelatin/aloe-vera/poly(ε-caprolactone) hybrid nanofibrous scaffold for application as skin substitutes. Mater. Sci. Eng. C.

[53] Zelepukin I.V., Griaznova O.Y., Shevchenko K.G., Ivanov A.V., Baidyuk E.V., Serejnikova N.B., Volovetskiy A.B., Deyev S.M., Zvyagin A.V. (2022). Flash drug release from nanoparticles accumulated in the targeted blood vessels facilitates the tumour treatment. Nat. Commun..

[54] Li L., Hao R., Qin J., Song J., Chen X., Rao F., Zhai J., Zhao Y., Zhang L., Xue J. (2022). Electrospun Fibers Control Drug Delivery for Tissue Regeneration and Cancer Therapy. Adv. Fiber Mater..

[55] Adepu S., Ramakrishna S. (2021). Controlled Drug Delivery Systems: Current Status and Future Directions. Molecules.

[56] Cezar C.A., Mooney D.J. (2014). Biomaterial-based delivery for skeletal muscle repair. Adv. Drug Deliv. Rev..

[57] Wang L., Cao L., Shansky J., Wang Z., Mooney D., Vandenburgh H. (2014). Minimally Invasive Approach to the Repair of Injured Skeletal Muscle With a Shape-memory Scaffold. Mol. Ther..

[58] Feng Y., Guo W., Hu L., Yi X., Tang F. (2022). Application of Hydrogels as Sustained-Release Drug Carriers in Bone Defect Repair. Polymers.

[59] Preethi A.M., Bellare J.R. (2021). Concomitant Effect of Quercetin- and Magnesium-Doped Calcium Silicate on the Osteogenic and Antibacterial Activity of Scaffolds for Bone Regeneration. Antibiotics.

[60] Xing Z.-C., Meng W., Yuan J., Moon S., Jeong Y., Kang I.-K. (2012). In Vitro Assessment of Antibacterial Activity and Cytocompatibility of Quercetin-Containing PLGA Nanofibrous Scaffolds for Tissue Engineering. J. Nanomater..

[61] Khil M.S., Cha D.I., Kim H.Y., Kim I.S., Bhattarai N. (2003). Electrospun nanofibrous polyurethane membrane as wound dressing. J. Biomed. Mater. Res. Part B Appl. Biomater. Off. J. Soc. Biomater. Jpn. Soc. Biomater. Aust. Soc. Biomater. Korean Soc. Biomater..

[62] Faraji S., Nowroozi N., Nouralishahi A., Shayeh J.S. (2020). Electrospun poly-caprolactone/graphene oxide/quercetin nanofibrous scaffold for wound dressing: Evaluation of biological and structural properties. Life Sci..

[63] Venugopal J., Ramakrishna S., Yang S., Leong K.-F., Du Z., Chua C.-K., Ma Z., He W., Yong T., Kotaki M. (2005). Biocompatible Nanofiber Matrices for the Engineering of a Dermal Substitute for Skin Regeneration. Tissue Eng..

[64] Jaisinghani R.N. (2017). Antibacterial properties of quercetin. Microbiol. Res..

[65] Wang S., Yao J., Zhou B., Yang J., Chaudry M.T., Wang M., Xiao F., Li Y., Yin W. (2018). Bacteriostatic Effect of Quercetin as an Antibiotic Alternative In Vivo and Its Antibacterial Mechanism In Vitro. J. Food Prot..

[66] Yang D., Wang T., Long M., Li P. (2020). Quercetin: Its Main Pharmacological Activity and Potential Application in Clinical Medicine. Oxidative Med. Cell. Longev..

[67] Yamaoka H., Asato H., Ogasawara T., Nishizawa S., Takahashi T., Nakatsuka T., Koshima I., Nakamura K., Kawaguchi H., Chung U.-I. (2006). Cartilage tissue engineering using human auricular chondrocytes embedded in different hydrogel materials. J. Biomed. Mater. Res. Part A.

[68] Ajmal G., Bonde G.V., Thokala S., Mittal P., Khan G., Singh J., Pandey V.K., Mishra B. (2019). Ciprofloxacin HCl and quercetin functionalized electrospun nanofiber membrane: Fabrication and its evaluation in full thickness wound healing. Artif. Cells Nanomed. Biotechnol..

[69] Vedakumari W.S., Ayaz N., Karthick A.S., Senthil R., Sastry T.P. (2017). Quercetin impregnated chitosan–fibrin composite scaffolds as potential wound dressing materials—Fabrication, characterization and in vivo analysis. Eur. J. Pharm. Sci..

[70] Lakhanpal P., Rai D.K. (2007). Quercetin: A versatile flavonoid. Internet J. Med. Update.

